# The Potential of Induced Pluripotent Stem Cells to Advance the Treatment of Pancreatic Ductal Adenocarcinoma

**DOI:** 10.3390/cancers13225789

**Published:** 2021-11-18

**Authors:** Ricki T. Krog, Noel F. C. C. de Miranda, Alexander L. Vahrmeijer, Nigel G. Kooreman

**Affiliations:** 1 Department of Surgery, Leiden University Medical Center, 2333 ZA Leiden, The Netherlands; R.T.Krog@lumc.nl (R.T.K.); A.L.Vahrmeijer@lumc.nl (A.L.V.); 2 Department of Pathology, Leiden University Medical Center, 2333 ZA Leiden, The Netherlands; N.F.de_Miranda@lumc.nl

**Keywords:** pancreatic ductal adenocarcinoma, PDAC, induced pluripotent stem cells, iPSCs, cancer models, cancer vaccine, cancer therapy, immunotherapy, microenvironment

## Abstract

**Simple Summary:**

Despite improvements in the treatment of several cancer types, the extremely poor prognosis of pancreatic cancer patients has remained unchanged over the last decades. Therefore, new therapeutic regimens for pancreatic cancer are highly needed. In this review, we will discuss the potential of induced pluripotent stem cells (iPSCs) to generate representative pancreatic cancer models that can aid the development of novel diagnostics and therapeutic strategies. Furthermore, the potential of iPSCs as pancreatic cancer vaccines or as a basis for cellular therapies will be discussed. With promising preclinical results and ongoing clinical trials, the potential of iPSCs to further the treatment of pancreatic cancer is being explored and, in turn, will hopefully provide additional therapies to increase the poor survival rates of this patient population.

**Abstract:**

Advances in the treatment of pancreatic ductal adenocarcinoma (PDAC) using neoadjuvant chemoradiotherapy, chemotherapy, and immunotherapy have had minimal impact on the overall survival of patients. A general lack of immunogenic features and a complex tumor microenvironment (TME) are likely culprits for therapy refractoriness in PDAC. Induced pluripotent stem cells (iPSCs) should be explored as a means to advance the treatment options for PDAC, by providing representative in vitro models of pancreatic cancer development. In addition, iPSCs could be used for tailor-made cellular immunotherapies or as a source of tumor-associated antigens in the context of vaccination.

## 1. Introduction

Pancreatic ductal adenocarcinoma (PDAC) is the most common type of pancreatic cancer, accounting for more than 90% of all cases [1]. PDAC has an extremely poor survival rate, of approximately 9% at five years, which has remained stable in the last decades despite improvements in the treatment of other cancer types [1,2]. Alarmingly, pancreatic cancer is predicted to become the second most common cause of cancers by 2030 in the United States [3]. For a long time, the standard treatment of PDAC consisted of chemotherapy or radiotherapy as a monotherapy. In recent years, this has changed towards slightly more efficient combined neoadjuvant treatments followed by surgical resection. Surgery is the only curative option of treatment for PDAC patients, but it applies to fewer than 20% of patients that are diagnosed in the early stage of disease prior to locally advanced, borderline unresectable, or metastatic disease [4]. Limited knowledge of both clinical symptoms and reliable biomarkers for precancerous lesions, such as pancreatic intraepithelial neoplasm (PanIN) [5], intraductal papillary mucinous neoplasm (IPMN) (reviewed in [6]), and acinar-to-ductal metaplasia (ADM) [7,8], are major obstacles for early diagnosis [9]. The lack of suitable models reflecting early stages of tumorigenesis of PDAC is an important contributor to the absence of diagnostic tools. The engraftment of PDAC cell lines or tumors in immunocompromised mice solely represents advanced disease stages and fails to provide means to develop strategies to halt PDAC development [10,11,12,13,14]. To address this, novel models are not only required to provide insights into disease progression but also to be a tool for the discovery of new therapeutic targets and improved drug screening [15,16]. Additionally, as previously stated, classical cancer models have major limitations representing the complex and constantly altering tumor microenvironment (TME) [17]. The PDAC TME consists of a large stromal component made up of an extracellular matrix, pancreatic stellate cells, cancer-associated fibroblasts (CAFs), and immune cells, with a majority of pro-tumorigenic cells such as myeloid-derived suppressor cells, tumor-associated M2-macrophages (TAMs), and regulatory T (T_reg_) cells; on the other hand, there are only a few anti-cancer effector cells such as M1 macrophages and effector CD4^+^ and CD8^+^ T cells. Establishment of complex multicellular models that represent the PDAC TME could open up avenues to test therapeutic interventions.

In recent years, we have seen cancer immunotherapies, in particular immune checkpoint inhibitors, redefine the potential of cancer treatments as they have produced remarkable improvements in the clinical outcome of patients with various cancer types [18,19,20,21]. However, these immunotherapies are mainly applicable to strongly immunogenic cancers with a high mutational burden and an associated TME with inflammatory features [22]. The large majority of PDAC patients have a low mutational burden and, as mentioned above, typically have a strongly immunosuppressive TME, which contributes to reduced drug responses [23,24,25,26]. Accordingly, most PDAC patients are refractory to checkpoint blockade [22]. Nevertheless, recent studies have demonstrated the existence of tumor-specific T cells in cancers with a low mutational burden, including PDAC [27,28,29,30], which supports the continuous attention to new immunotherapy strategies for this disease (reviewed in [31]). In fact, several clinical trials are currently recruiting PDAC patients for the treatment with at least one immunotherapeutic agent (Table S1 (ClinicalTrials.gov, 16 November 2021)). The focus of these clinical trials is mainly on new combinatorial therapies, including checkpoint inhibitors. However, PDAC vaccines and a cellular immunotherapy are also being investigated clinically (Table S1 (ClinicalTrials.gov, 16 November 2021)). Additionally, other immunotherapeutic strategies such as targeting the WNT-signaling pathway to enhance the PDAC-directed immune responses seems promising in PDAC patients with lymph node metastasis (reviewed in [32]) [33].

Here, we discuss induced pluripotent stem cell (iPSC)-based models as a promising tool to model PDAC development and treatment responses. Aside from their use as models, they also hold great potential for novel immunotherapies.

## 2. The Potential of Induced Pluripotent Stem Cells (iPSCs)

iPSCs provide excellent possibilities for the modeling of PDAC and serve as a unique source of tumor-associated antigens for whole-cell cancer vaccines and immune cells for adoptive cellular immunotherapies. Pluripotent stem cells hold stemness, which is defined as the ability to proliferate indefinitely while maintaining pluripotency. Embryonic stem cells (ESCs) are a classic example of this capability and are capable of differentiating into all of the three embryonic germ layers, thus giving rise to all adult cell types [34,35]. Since the isolation of ESCs, scientists have mainly focused on their extraordinary potential for (personalized) regenerative medicine. Takahashi and Yamanaka demonstrated in 2006 that defined culture conditions, including the exogenous supply of four transcription factors (Yamanaka factors) MYC, OCT3/4, SOX2, and KLF4, were capable of reprogramming differentiated mouse embryonic and adult fibroblast cells into cells with characteristics of ESCs [36]. These dedifferentiated pluripotent cells were termed induced pluripotent stem cells (iPSCs). This discovery was immediately followed by the induction of human iPSCs from terminally differentiated cells, and in subsequent years various methods have been developed to generate iPSCs from a variety of somatic cells (reviewed in [37,38]) [39,40,41,42,43,44,45,46,47,48,49,50]. These accomplishments led to the exploration of the potential of iPSCs for disease modeling and treatment of various diseases [39,40]. iPSCs share a number of characteristics with ESCs with almost identical gene expression and epigenetic status and possess the unique feature of stemness [51,52,53,54]. Furthermore, ethical issues associated with human ESCs are avoided; thus, iPSCs expand the range of applications in which stem cells can be exploited. These applications include basic cell biology, disease models, and drug discovery and screening. Furthermore, iPSCs provide the potential for new clinical applications and can serve as the basis for (off-the-shelf) cancer immunotherapies. The pros and cons of iPSCs related to cancer immunotherapies and models are listed in Table 1.

## 3. iPSC-Based PDAC Models and Their Potential for Disease Modeling

Around 20% of PDAC patients in the United States are diagnosed with localized disease and are therefore eligible for surgical resection [2,4]. However, the majority of patients are diagnosed with locally advanced or metastasized disease at diagnosis, which leaves these patients with a poor survival rate [2]. Limited knowledge of both clinical symptoms and biomarkers in the early stages of PDAC are major obstacles for early disease stage diagnosis [9]. A number of genomic alterations have been associated with PDAC, but our understanding of their precise role in the onset and progression of disease is limited as the genomic studies associated with disease progression are sparse due to the lack of suitable models. For example, PDAC-derived xenografts have been established in immunocompromised mice by using tumor tissues or cell lines [10,11,12,13,14]. These models solely reflect the invasive stages of PDAC and are not suitable for studies on the onset and early stages of PDAC. Novel models providing a better understanding of the biological processes at the basis of tumorigenesis are essential to improve diagnostics. To address this, iPSCs can provide a source of cells that better reflect the early stages of malignant transformation in PDAC.

iPSC-derived cancer-initiating cells have previously been reported for the establishment of xenograft models that reflect the malignant transformation in PDAC [56,71]. Mouse iPSCs from healthy cells have been differentiated in a controlled manner into PDAC progenitor cells [56]. Xenograft models originating from these cells were able to give rise to precancerous lesions, including ADM and PanIN, as well as invasive PDAC. Exploiting a different approach, Kim et al. (2013) hypothesized that a subset of iPSCs induced from human PDAC cells would result in malignant iPSC lines, capable of undergoing early developmental stages of PDAC after engraftment into mice [58]. One of the generated iPSC lines carried a *KRAS^G12D^* mutation and a deletion of *CDKN2A*. The oncogenic *KRAS* mutations are the most frequently detected oncogenic alteration in PDAC, being observed in >90% of patients [72,73,74]. *CDKN2A* is a tumor suppressor in PDAC and has been described as being inactivated in approximately 50% of patients [75,76]. Xenografts originating from the *KRAS^G12D^ CDKN2A^−\−^* iPSC line gave rise to PanIN-like lesions followed by progression to invasive PDAC [58]. iPSC-based xenograft PDAC models originating from malignant cells demonstrate the potential of iPSCs to provide insights into PDAC onset and progression, including the identification of potential biomarkers for early diagnosis of PDAC. Another application where iPSCs might improve PDAC-modeling is the generation of iPSC-derived organoids containing different cell populations. iPSCs can be committed to a differentiation into the pancreatic exocrine lineage for the generation of acinar and ductal cells and, thus, provide great organoid-modeling possibilities for PDAC [55,57,77,78]. PDAC can develop from both acini and ducts, however knowledge on how these two cells of origin impact cell progression is scarce [79]. Two studies recently assessed how the PDAC oncogenes *KRAS* and *GNAS* individually affect the growth and progression of PDAC in vitro and in vivo after engraftment of iPSC-derived acinar and ductal organoids in immunocompromised mice [55,57]. Both KRAS^G12D^-mutated acinar and ductal organoids displayed proliferation in vivo, although the more invasive lesions were generated from acinar organoids. Phenotypically, both oncogenic alterations caused IPMN-like lesions in vivo. Furthermore, PanIN lesions and different stages of PDAC-like tumor formation were observed in xenografts from KRAS^G12D^-mutated ductal and acinar organoids. In vitro, KRAS^G12D^-mutated ductal organoids displayed epithelial-to-mesenchymal transition (EMT), which have been suggested to play a role in early tumor formation, metastasis, and chemoresistance in PDAC [80,81,82]. In contrast, GNAS^R201C/H^ induced cystic growth in vitro in ductal organoids and to a lesser extend in acinar organoids. These iPSC-derived models provide vital knowledge of the malignant potential of different oncogenes in PDAC. Furthermore, the models provide great opportunities for in-depth assessment of early-stage disease development and progression.

In addition to the above-mentioned applications of iPSCs for disease modeling, iPSCs can also be differentiated into non-malignant cells of the TME. This opens up avenues for the development of complex multicellular models to test therapeutic interventions. For example, TAMs are thought to play an important role in PDAC tumorigenesis and may constitute promising clinical targets [26]. Macrophage models for drug discovery have so far been dependent on a limited source of monocytes derived from PBMCs or animal bone marrow, which has limited the generation of models representative of tissue-resident macrophages (reviewed in [83]). Gutbier and colleagues established a method for controlled large-scale iPSC-derived tissue-resident-resembling macrophages for efficient drug screening and discovery [84]. Genetic manipulation of these iPSC-derived macrophages can be conducted to obtain the desired macrophage subtype. Additionally, cancer-initiating cells originating from iPSCs from healthy cells can also be differentiated into CAFs and vascular endothelial-like cells in vivo [59,60,61]. Particularly, CAFs have been implicated as important players in the tumorigenesis of PDAC (reviewed in [85]) [86,87]. CAFs constitute a promising therapeutic target in PDAC and several therapeutic strategies have been investigated preclinically and clinically (reviewed in [88,89]). The versatility of iPSCs to generate a variety of cells from the TME can support the development of models that include various cell types [90]. Additionally, the directed differentiation towards a cell line of interest shows the potential of iPSC-derived models for drug screening at the molecular level.

iPSC-based xenografts and organoids provide excellent innovative possibilities for the modeling of PDAC, especially to study precancerous lesions and the development of this disease. Furthermore, the potential of iPSCs as a source for a variety of cells provides an opportunity for the establishment of multicellular models that better represent the PDAC TME. However, iPSC-based PDAC models are still in the early phase and further research is needed to fully exploit their potential.

## 4. iPSCs as a Cell-Based Immunotherapy

PDAC is characterized by a low mutational burden and, consequently, a low amount of neoantigens are generated for spontaneous antitumor immune responses by the adaptive immune system [22]. Additionally, the highly immunosuppressive TME of PDAC further contributes to its poor immunogenic character. A classical way of stimulating a specific immune response is by antigen vaccination. Therapeutic cancer vaccines aim to stimulate antitumor immunity, e.g., by supporting the activation of cancer-specific CD8^+^ and CD4^+^ T cells. ESCs have been hypothesized to serve as an efficient source of antigens for cancer vaccines due to their immunogenicity and shared antigenic profile with cancer cells [91,92,93]. Similar to ESCs, iPSCs have an immunogenic potential and share antigens with PDAC cells, making them an attractive source of antigens for cancer vaccination (reviewed in [94]) [65,68,95]. Kooreman et al. have found that an iPSC-based cancer vaccine was capable of eliciting an immune response towards shared iPSC and cancer cell antigens in murine cancer models [65]. The vaccine consisted of autologous iPSCs and the toll-like receptor 9 (TLR9) agonist CpG, to enhance the immunostimulatory properties of the vaccine. In murine models of breast, lung, and skin cancer, this iPSC-based cancer vaccine elicited a potent humoral and cell-mediated immune response sufficient to prevent or limit tumor growth in vivo without any observed associated adverse effects [65]. In a mouse model of PDAC, the same vaccine induced protective immunity characterized by the expansion of effector and memory CD8^+^ T cells, CD4^+^ T cells (excluding T_reg_ cells), and B cells, while reducing the amount of T_reg_ cells in tumor-draining lymph nodes [68]. Furthermore, several cancer-signature peptide antigens, containing previously experimentally reported T cell epitopes, were identified in the vaccine in silico; this suggests the possibility for expansion of PDAC antigen-specific effector T cells [68]. This iPSC-based vaccine was proposed as a promising tool to be employed in an adjuvant context, in combination with conventional therapies [65]. Furthermore, this type of vaccination could hold promise in a prophylactic or (neo)adjuvant setting to stimulate the immune system in combination with other immunotherapies, such as immune checkpoint inhibitors, adaptive transfer of primed autologous T cells, chimeric antigen receptor (CAR) cells, or modulators of the immunosuppressive TME, by targeting the WNT-signaling pathway (reviewed in [32]) [33].

Prophylactic vaccines can play a role in the prevention of cancers caused by viruses, e.g., human papillomaviruses (HPVs) and the hepatitis B virus (HBV). To date, no prophylactic vaccines aiming at preventing non-viral-related cancers have been approved. Lu et al. (2020) reported an iPSC-based autologous prophylactic cancer vaccination regimen that was evaluated using the KPC mouse model of PDAC with spontaneous tumor development [67]. The PDAC driver mutations Kras^G12D^ and p53^R172H^ were introduced in murine iPSCs derived from healthy cells followed by controlled differentiation into PDAC progenitor cells. These cells were antigenically comparable to PDAC cells from the KPC mice and, therefore, serve as a good repertoire of PDAC-expressing antigens. The iPSC-induced PDAC progenitor cells were infected with a non-replicating oncolytic virus to enhance the immunogenicity of the final vaccine formulation. Importantly, the immunogenicity of the vaccine demonstrated a clinical impact by delaying tumor development and prolonging survival of the KPC mice vaccinated before tumor development. However, the vaccine failed to provide complete protection from tumor development in the mice. Upon vaccination, CD8^+^ T cell infiltration increased in the PDAC TME and an accumulation of central memory T cells was observed in the secondary lymph nodes. Furthermore, splenocytes from vaccinated mice, prior to tumor development, showed enhanced production of the proinflammatory cytokine IFNγ after ex vivo challenge with tumor-cell lines derived from the corresponding model. This iPSC-based autologous prophylactic cancer vaccination regimen demonstrates promising results in a mouse model of PDAC and further studies will clarify whether iPSC-based prophylactic PDAC vaccines have a potential for the prevention of pancreatic malignancies in at-risk individuals.

**Figure 1:**
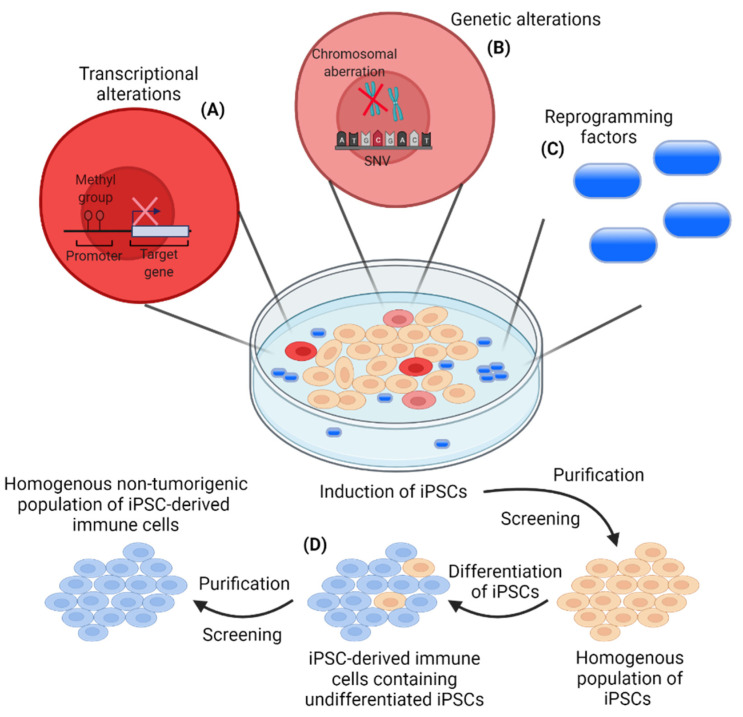
Risks associated with the induction of induced pluripotent stem cells (iPSCs) and the generation of a homogenous non-tumorigenic population of iPSC-derived immune cells. (**A**) Transcriptional alterations (aberrant DNA methylation) can occur in some iPSC clones during the induction of iPSCs leading to clones with a lower differential potential. This heterogenous iPSC induction, due to an improper epigenetic status of some iPSC clones, constitutes a major limitation in the induction of a homogenous population of iPSCs, which should be addressed by optimized reprogramming protocols; (**B**) Induction of iPSCs includes the risk of genetic alterations by the introduction of single nucleotide variants (SNVs) and/or chromosomal aberrations. The mutation rate has been demonstrated to be around 10 times lower for iPSCs compared to somatic cells. However, genetic alterations will occur and presents an issue in the induction of a homogenous population of iPSCs for further applications. The risk of genetic alterations, influencing the phenotype of a subpopulation of iPSCs, highlights the importance of efficient screening and purification of the induced iPSCs; (**C**) Induction of iPSCs is carried out with the use of reprogramming factors with a potential tumorigenic potential. This necessitates the efficient removal of the reprogramming factors from the final iPSC population prior to clinical applications; (**D**) Remnant undifferentiated iPSCs constitute a potential risk for tumor formation in patients. Efficient purification and screening must be conducted to avoid undifferentiated iPSCs in the final population of iPSC-derived immune cells for clinical applications.

The first clinical use of iPSC-based cancer vaccines is yet to be seen, but the above-mentioned preclinical studies demonstrate the potential of these vaccines to elicit anti-PDAC immune responses. In addition to PDAC therapy, iPSC-based antitumor vaccination could potentially serve as a promising universal approach in a broad spectrum of cancer types, including mesothelioma, breast cancer, and melanoma (reviewed in [96]) [65,97,98,99]. Tumorigenic properties of iPSCs necessitate lethal irradiation of the iPSCs prior to injection into patients to avoid a potential risk of tumor formation (reviewed in [100,101]) [65,68,102,103]. Additionally, care must be taken to avoid activity of remnant transcription factors used for the induction of the iPSCs (Figure 1). Three of the four Yamanaka factors, MYC, OCT3/4, and SOX2, are proto-oncogenes and are involved in tumorigenesis [93,104,105,106,107,108]; these transcription factors have been extensively reviewed elsewhere [109,110,111]. Efficient screening and purification must be carried out to secure a safe vaccine formulation before clinical implementation.

## 5. iPSC-Derived Immune Cells for Cancer Immunotherapies

Current approaches focusing on T cell immunotherapies, such as autologous T cell transfer, engineered T cell receptor (TCR) T cells, and CAR T cells, typically require autologous cell manufacturing for each individual patient. Therefore, there is an unmet need for innovative cell sources to broaden the application of cellular immunotherapies [112]. iPSCs can be a permanent source of various immune cells, which can be genetically modified for optimal therapeutic features. Of note, cells terminally differentiated from iPSCs are proposed to lose the immunogenic features associated with undifferentiated iPSCs [95]. A manufacturing system has been developed for the consistent differentiation and expansion of functional iPSC-derived NK cells from a single source of iPSCs [62]. These NK cells delayed solid tumor progression in vivo and enhanced the migration of T cells into the tumor area [62], demonstrating the potential of iPSCs as a source of adoptive cellular immunotherapies.

CAR cells serve as an innovative approach to support the recognition and killing of cancer cells in a human leukocyte antigen (HLA)-independent manner, thereby circumventing tumor escape mechanisms associated with defects in antigen presentation (reviewed in [113]). CD19-directed CAR T cells have been approved in recent years for the treatment of a subset of patients with relapsed/refractory B cell cancers, and the CAR technology holds great promise to expand into treatments of more cancer types (reviewed in [114,115]). Combining iPSC-based T cells with the CAR technology for the generation of CD19-specific CAR T cells demonstrated clinical efficacy in a xenograft cancer model [69]. This proof-of-concept of iPSC-based CAR T cells was followed by the induction of iPSC-based CAR NK cells and CAR macrophages [66,70], thereby demonstrating the potential of iPSCs to expand the range of effector cell types available for CAR cell therapies, including a potentially easily accessible and unlimited supply of the CAR cells from a single source of iPSCs. Of note, the immune cell of choice affects the degree of adverse effects of iPSC-derived cellular immunotherapies. iPSC-derived CAR NK cells were demonstrated to induce less toxicity in vivo compared to CAR T cells [66]. While CAR NK and T cells showed similar antitumor responses, the CAR NK cell therapy improved survival of tumor-bearing mice due to limited cytokine release and therapy-associated pathological damage [66], thereby demonstrating that the effector cell of choice for CAR engineering is crucial for the clinical outcome of CAR cell therapies. To address the adverse effects of CAR cell therapies, alloreactivity of the endogenous CAR TCR and/or allorejection must be taken into consideration (reviewed in [116]). A path to address this is by using hypoimmunogenic iPSCs that lack the expression of HLA class I and II genes [63]. Derivates of these hypoimmunogenic iPSCs evade immune rejection in allogeneic recipients [63], demonstrating the potential of hypoimmunogenic iPSCs as a future universal donor for cellular immunotherapies.

Tumor escape from CAR cells due to the complex immunosuppressive TMEs, as observed in PDAC, remains an obstacle for CAR cell therapies, and strategies to overcome this have been proposed [117]. One strategy is converting the TME into a more proinflammatory environment. Macrophages are a source of cytokines and chemokines, and, consequently, have a great impact on the characteristics of the TME as immunosuppressive (TAMs) or proinflammatory (M1 macrophages). Zhang et al. (2020) demonstrated a platform for a consistent source of iPSC-derived CAR macrophages from human PBMCs [70]. iPSC-derived macrophages have been genetically engineered with CARs to facilitate antigen-dependent phagocytosis of cancer cells and have been demonstrated to direct the TME towards a more proinflammatory environment [64,70,118,119]. In another study, Klichinsky et al. (2020) genetically engineered CAR macrophages with the transduction of CARs in a replication-incompetent adenoviral vector [64]. Aside from the delivery of the CARs by the viral vector, the exposure to the virus led to the induction of a proinflammatory M1 phenotype of the CAR macrophages. These iPSC-based M1 CAR macrophages could, at the same time, provide the means to kill target cells and shape the state of the TME.

Despite being in the early stages, iPSC-derived NK cells (ClincalTrials.gov, Identifier: NCT03841110) and NKT cells (jrct.niph.go.jp, Trial ID: jRCT2033200116) are currently being clinically investigated in patients with advanced solid tumors. It is worth noting that PDAC patients will be included for monotherapy with the iPSC-derived NK cells or as a combinatorial therapy with immune checkpoint inhibitors. With the increasing availability of promising preclinical data, it is plausible that additional clinical trials will be initiated in the near future. However, before clinical implementation, all safety issues must be thoroughly addressed (discussed in Section 6). Taken together, iPSCs serve as a promising approach for a novel source of a variety of immune cells for adoptive cellular immunotherapies.

## 6. The Importance of Controlled Reprogramming and Differentiation of iPSCs

The genetic source of the generated iPSCs is the major factor of variation between different populations of iPSCs [54]. This makes choosing the optimal source for the generation of iPSCs important for the final product. In addition, controlled reprogramming is crucial and should be geared towards the generation of a homogeneous population of undifferentiated iPSCs. This is important, as heterogenicity in an iPSC population possesses the risk of uncontrolled differentiation potential of the iPSCs [120]. Unique transcriptional alterations can be introduced in the iPSCs by the specific reprogramming system of choice [54,121]. Reprogramming can lead to aberrant DNA methylation patterns in iPSCs, leading to clones with lower differentiation potential (Figure 1) [120]. To avoid heterogeneity introduced by epigenetic abnormalities, procedures have been established to introduce global DNA demethylation of the iPSCs (reviewed in [122]). Furthermore, culturing iPSCs increases the risk of genetic alterations by the introduction of single nucleotide variants (SNVs) and/or chromosomal aberrations (Figure 1) [123,124]. In general, DNA repair mechanisms ensure genetic stability. However, errors will occur at a certain mutation rate. While 14 SNVs have been estimated to be introduced in somatic cells per generation, the mutation rate has been demonstrated to be around 10 times lower for iPSCs [124]. Nevertheless, the lower mutation rate does not rule out the risk of heterogeneity introduced by genetic alterations.

In addition to the risks in the reprogramming of iPSCs, remnant undifferentiated iPSCs in the final clinical product constitute a risk for tumor formation in patients (reviewed in [100,101]), which has been demonstrated in vivo after the engraftment of undifferentiated iPSCs in mice (Figure 1) [102,103]. To avoid this risk, several approaches to eliminate remaining undifferentiated iPSCs have been reported, including immunologic targeting of the iPSCs or cell sorting by antibodies [125,126,127,128], small-molecule inhibitors [128,129,130,131], or incorporation of a suicide gene into the iPSCs [132]. The reported purification methods have been extensively reviewed elsewhere [122,133].

## 7. Conclusions

PDAC has an extremely poor prognosis, which has remained unchanged over the last decades. Therefore, novel strategies for the modeling and treatment of PDAC are highly needed. iPSCs provide innovative options for the modeling of precancerous lesions as well as the early development and progression of PDAC, which can improve knowledge of clinical-relevant biomarkers early after the onset of disease. Furthermore, iPSCs constitute a novel consistent source of a variety of cells for studying this disease in complex systems. In recent years, increased attention has been giving to the TME due to its important role in the success of immunotherapies (reviewed in [134]). iPSC-based multicellular models can potentially reflect the complexity of the PDAC TME and, thereby, open up avenues to improve drug screening and test new therapies. Nevertheless, further research is needed to fully exploit the potential of iPSC-based PDAC models and, eventually, serve as the basis for clinical breakthroughs, e.g., by improved diagnostics and drug screenings. Undifferentiated iPSCs also provide unique possibilities for future PDAC vaccines. Potentially, vaccination with iPSCs could serve as a potent (neo)adjuvant treatment that enhances antitumor immune responses. In contrast to whole-cell vaccination, modern subunit or mRNA vaccines consist of a limited amount of well-defined antigens or mRNAs encoding the antigens, respectively. The whole-cell cancer vaccine strategy holds the advantage of including a broad range of antigens, including unknown cancer antigens, in the vaccine formulation. Additionally, clinically relevant immunogenic epitopes are possibly shared between patients, allowing for an off-the-shelf iPSC vaccine product, thus eliminating the need for costly and labor-intensive personalized vaccine formulations.

Most PDAC patients are refractory to immune checkpoint inhibitors, which is likely due to a low mutational burden and strongly immunosuppressive TME, even though neoantigen-specific effector T cells have been demonstrated in the TME of PDAC patients [29,30]. A rationale for this might be found in cancer patients with non-small cell lung carcinoma (NSCLC) that are unresponsive to immune checkpoint inhibitors. Recent studies in these patients have shown that the ineffective antitumor responses seem to be associated with improper maturation of TCF1^+^ CD8^+^ T cells in the tumor-draining lymph nodes as well as in the TME [135,136]. Likely, the TME is responsible for the lack of proper maturation of these T cells as additional cytokine support restores the differentiation of antitumor effector T cells in the tumor-draining lymph nodes [135]. If a similar process occurs in PDAC patients, iPSC-based vaccination might address this issue by generating a pool of antitumor effector and memory T cells outside of the TME, thereby allowing the T cells to fully mature.

The pluripotency and indefinite proliferation capacity of iPSCs allow these cells to serve as a consistent cell source for various types of adoptive cellular immunotherapies. Additionally, hypoimmunogenic derivates of iPSCs have been reported, thus providing great potential for the generation of an iPSC line as a universal donor for off-the-shelf cellular immunotherapies [63]. Consequently, these cells might eliminate the need for complicated, inefficient, and costly autologous cell manufacturing, and the cell product could potentially be available to the patient at the time of diagnosis. Furthermore, iPSC-derived immune cells, specifically those affecting or targeting the TME of PDAC patients, hold great promise for inducing more favorable conditions for antitumor responses. Additionally, other immunotherapies modulating the TME, such as checkpoint inhibitors and tankyrase inhibitors targeting the WNT-signaling pathway, could be employed in combination with iPSC-based immunotherapies to enhance the PDAC-directed immune responses.

A note of caution is warranted regarding the reprogramming and differentiation of iPSCs. First, iPSCs possess the risk of transcriptional and genetic alterations. Therefore, controlled induction of iPSCs is essential to ensure a homogenous population for further applications. Second, to reduce potential risks of tumorigenesis, the final cell product of iPSC-derivates should include a pure population of differentiated cells without remnant undifferentiated iPSCs that could form teratomas.

With the promising preclinical results for iPSC-based immunotherapies and two clinical trials ongoing, including one with PDAC patients, it seems plausible to expect more clinical trials to be initiated in the near future. Hopefully, these trials will advance the treatment options for PDAC patients and, in turn, increase their survival rates.

## Figures and Tables

**Table 1:** Pros and cons of induced pluripotent stem cells (iPSCs) related to cancer immunotherapies and models.

	Pros +		Cons −	
**Models** [55,56,57,58,59,60,61]	+	Insight into disease onset and development	−	Risk of transcriptional and genetic alterations during iPSC induction
	+	Reliable cell source for multicellular models	−	Heterogeneity within iPSC populations
**Immunotherapeutic approaches** [62,63,64,65,66,67,68,69,70] *(ClincalTrials.gov, Identifier: NCT03841110* *jrct.niph.go.jp, Trial ID: jRCT2033200116)*	+	iPSCs are immunogenic and express tumor-associated antigens	−	Oncogenic potential of iPSCs reprogramming factors
	+	Reliable cell source for off-the-shelf cellular therapies	−	Risk of transcriptional and genetic alterations during iPSC induction
	+	Large-scale cell production	−	Heterogeneity within iPSC populations

## Supplementary Materials

The following are available online at https://www.mdpi.com/article/10.3390/cancers13225789/s1, Table S1: Clinical trials currently recruiting PDAC patients for the treatment with at least one immunotherapy agent.
