# Supplementary material for: The Potential of Induced Pluripotent Stem Cells to Advance the Treatment of Pancreatic Ductal Adenocarcinoma
Source: Cancers (Basel). 2021 Nov 18;13(22):5789. doi: 10.3390/cancers13225789 (PMC8616212; doi:10.3390/cancers13225789)
Supplement: Supplementary file 1 [file cancers-13-05789-s001.zip › cancers-1437653-supplementary.pdf]

Table S1: Clinical trials currently recruiting PDAC patients for the treatment with at least one immuno-therapy agent.

ClinicalTrials.gov Search Results 11/16/2021

	Title	Status	Study Results	Conditions	Interventions	Locations
1	A Beta-only IL-2 ImmunoTherapY (ABILITY) Study	Recruiting	No Results Available	•Advanced Solid Tumor<br>•Unresectable Solid Tumor<br>•Melanoma<br>•Renal Cell Carcinoma<br>•Sarcoma<br>•Triple Negative Breast Cancer<br>•Pancreatic Ductal Adenocarcinoma<br>•Non-Small Cell Lung Cancer Squamous<br>•Non-Small Cell Lung Cancer Non-squamous<br>•Colorectal Cancer<br>•and 12 more	•Drug: MDNA11 Monotherapy<br>•Drug: MDNA11 in combination with checkpoint inhibitor	•Chris O'Brien Lifehouse, Camperdown, New South Wales, Australia<br>•Scientia Clinical Research, Randwick, New South Wales, Australia<br>•Gallipoli Medical Research Foundation, Greenslopes, Queensland, Australia<br>•ICON Cancer Center, South Brisbane, Queensland, Australia<br>•Cabrini Research, Malvern, Victoria, Australia
2	Study of INCA 0186 in Subjects With Advanced Solid Tumors	Recruiting	No Results Available	•Advanced Solid Tumors<br>•Squamous Cell Carcinoma of the Head and Neck (SCCHN)<br>•Gastrointestinal (GI) Malignancies	•Drug: INCA00186<br>•Drug: Retifanlimab<br>•Drug: INCB106385	•The Angeles Clinic and Research Institute, Los Angeles, California, United States<br>•Carolina Bio-Oncology Institute, Pllc, Huntersville, North Carolina, United States
3	Lenvatinib and Pembrolizumab Maintenance Therapy for the Treatment of Patients of Advanced Unresectable Pancreatic Cancer	Recruiting	No Results Available	•Advanced Pancreatic Ductal Adenocarcinoma<br>•Stage II Pancreatic Cancer AJCC v8<br>•Stage IIA Pancreatic Cancer AJCC v8<br>•Stage IIB Pancreatic Cancer AJCC v8<br>•Stage III Pancreatic Cancer AJCC v8<br>•Stage IV Pancreatic Cancer AJCC v8<br>•Unresectable Pancreatic Ductal Adenocarcinoma	•Drug: Lenvatinib Mesylate<br>•Biological: Pembrolizumab	•City of Hope Medical Center, Duarte, California, United States
4	A Study of ELI-002 in Subjects With KRAS Mutated Pancreatic Ductal Adenocarcinoma (PDAC) and Other Solid Tumors	Recruiting	No Results Available	•Minimal Residual Disease<br>•KRAS G12D<br>•KRAS G12R<br>•NRAS G12D<br>•NRAS G12R<br>•Pancreatic Ductal Adenocarcinoma<br>•Colorectal Cancer<br>•Non-small Cell Lung Cancer<br>•Ovarian Cancer<br>•Cholangiocarcinoma<br>•Bile Duct Cancer<br>•Gallbladder Carcinoma	•Drug: ELI-002 2P	•City of Hope, Duarte, California, United States<br>•University of California Los Angeles, Los Angeles, California, United States<br>•University of Colorado, Aurora, Colorado, United States<br>•University of Iowa, Iowa City, Iowa, United States<br>•Massachusetts General Hospital, Boston, Massachusetts, United States<br>•Henry Ford Cancer Institute, Detroit, Michigan, United States<br>•Washington University School of Medicine, Saint Louis, Missouri, United States<br>•Northwell Health, Lake Success, New York, United States<br>•Memorial Sloan Kettering Cancer Center, New York, New York, United States<br>•Tennessee Oncology - Centennial Clinic, Nashville, Tennessee, United States<br>•The University of Texas MD Anderson Cancer Center, Houston, Texas, United States

	Title	Status	Study Results	Conditions	Interventions	Locations
5	Study to Evaluate the Safety and Efficacy of Treatment With NLM-001 and Standard Chemotherapy Plus Zalifrelimab in Patients With Advanced Pancreatic Cancer	Recruiting	No Results Available	•Pancreatic Ductal Adenocarcinoma	•Drug: Gemcitabine<br>•Drug: Nab paclitaxel<br>•Drug: NLM-001<br>•Drug: Zalifrelimab	•Hospital Universitario Virgen De La Victoria, Málaga, Andalucía, Spain<br>•Hospital Universitario Miguel Servet, Zaragoza, Aragón, Spain<br>•Hospital Universitari Vall d'Hebron, Barcelona, Cataluña, Spain<br>•Hospital Universitario Donostia, San Sebastián, País Vasco, Spain
6	Multi-agent Low Dose Chemotherapy GAX-CI Followed by Olaparib and Pembro in Metastatic Pancreatic Ductal Cancer.	Recruiting	No Results Available	•Metastatic Pancreatic Cancer	•Drug: Nab-paclitaxel<br>•Drug: Gemcitabine<br>•Drug: Cisplatin<br>•Drug: Irinotecan<br>•Drug: Capecitabine<br>•Drug: Pembrolizumab<br>•Drug: Olaparib	•Sidney Kimmel Comprehensive Cancer Center, Baltimore, Maryland, United States
7	PANFIRE-3 Trial: Assessing Safety and Efficacy of Irreversible Electroporation (IRE) + Nivolumab + CpG for Metastatic Pancreatic Cancer	Recruiting	No Results Available	•Pancreatic Cancer<br>•Metastatic Pancreatic Cancer	•Device: Irreversible Electroporation (IRE)<br>•Drug: Nivolumab<br>•Drug: Toll-Like Receptor 9	•Amsterdam University Medical Centre (location VUmc), Amsterdam, North-Holland, Netherlands
8	Niraparib and Dostarlimab for the Treatment of Germline or Somatic BRCA1/2 and PALB2 Mutated Metastatic Pancreatic Cancer	Recruiting	No Results Available	•Metastatic Pancreatic Ductal Adenocarcinoma<br>•Stage IV Pancreatic Cancer AJCC v8	•Biological: Dostarlimab<br>•Drug: Niraparib	•Mayo Clinic in Arizona, Scottsdale, Arizona, United States<br>•Mayo Clinic in Florida, Jacksonville, Florida, United States<br>•Mayo Clinic in Rochester, Rochester, Minnesota, United States
9	Pooled Mutant KRAS-Targeted Long Peptide Vaccine Combined With Nivolumab and Ipilimumab for Patients With Resected MMR-p Colorectal and Pancreatic Cancer	Recruiting	No Results Available	•Colorectal Cancer<br>•Pancreatic Cancer	•Drug: KRAS peptide vaccine<br>•Drug: Nivolumab<br>•Drug: Ipilimumab	•Sidney Kimmel Comprehensive Cancer Center, Baltimore, Maryland, United States
10	Nivolumab in Combination With Chemotherapy Pre-Surgery in Treating Patients With Borderline Resectable Pancreatic Cancer	Recruiting	No Results Available	•Borderline Resectable Pancreatic Adenocarcinoma<br>•Resectable Pancreatic Ductal Adenocarcinoma	•Drug: Fluorouracil<br>•Drug: Irinotecan<br>•Drug: Irinotecan Hydrochloride<br>•Drug: Leucovorin<br>•Drug: Leucovorin Calcium<br>•Biological: Nivolumab<br>•Drug: Oxaliplatin<br>•Procedure: Therapeutic Conventional Surgery	•UCLA / Jonsson Comprehensive Cancer Center, Los Angeles, California, United States
11	Maintenance With OSE2101 Plus FOLFIRI, or FOLFIRI After FOLFIRINOX-based Induction Therapy in Locally Advanced or Metastatic Pancreatic Ductal Adenocarcinoma	Recruiting	No Results Available	•Pancreatic Ductal Adenocarcinoma<br>•Locally Advanced Cancer<br>•Metastatic Cancer	•Drug: FOLFIRI<br>•Drug: OSE2101	•Clinique de l'Europe, Amiens, France<br>•Hôpital Sud CHU Amiens, Amiens, France<br>•CH Beauvais, Beauvais, France<br>•CHRU Jean Minjoz, Besançon, France<br>•Clinique Tivoli Ducos, Bordeaux, France<br>•CHU Morvan, Brest, France<br>•GHPSO Site de Creil, Creil, France<br>•Hôpital Henri Mondor, Créteil, France<br>•Centre Georges François Leclerc, Dijon, France<br>•CHU Dijon, Dijon, France<br>•and 18 more

	Title	Status	Study Results	Conditions	Interventions	Locations
12	Trial of Neoadjuvant and Adjuvant Nivolumab and BMS-813160 With or Without GVAX for Locally Advanced Pancreatic Ductal Adenocarcinomas.	Recruiting	No Results Available	• Locally Advanced Pancreatic Ductal Adenocarcinoma (PDAC)<br>• Pancreatic Ductal Adenocarcinoma	• Radiation: Stereotactic Body Radiation (SBRT)<br>• Drug: Nivolumab<br>• Drug: CCR2/CCR5 dual antagonist<br>• Drug: GVAX	• Sidney Kimmel Comprehensive Cancer Center, Baltimore, Maryland, United States
13	Study of Pembrolizumab With or Without Defactinib Following Chemotherapy as a Neoadjuvant and Adjuvant Treatment for Resectable Pancreatic Ductal Adenocarcinoma	Recruiting	No Results Available	• Resectable Pancreatic Ductal Adenocarcinoma (PDAC)<br>• Pancreatic Ductal Adenocarcinoma	• Drug: Pembrolizumab<br>• Drug: Defactinib	• Samuel Oschin Cancer Center at Cedars-Sinai, Los Angeles, California, United States<br>• Sidney Kimmel Comprehensive Cancer Center, Baltimore, Maryland, United States
14	A Phase I/Ib Study of NZV930 Alone and in Combination With PDR001 and /or NIR178 in Patients With Advanced Malignancies.	Recruiting	No Results Available	• Non-small Cell Lung Cancer (NSCLC)<br>• Triple Negative Breast Cancer (TNBC)<br>• Pancreatic Ductal Adenocarcinoma (PDAC)<br>• Colorectal Cancer Microsatellite Stable (MSS)<br>• Ovarian Cancer<br>• Renal Cell Carcinoma (RCC)<br>• Metastatic Castration Resistant Prostate Cancer (mCRPC)	• Other: NZV930<br>• Other: PDR001<br>• Drug: NIR178	• H Lee Moffitt Cancer Center and Research Institute Inc, Tampa, Florida, United States<br>• University of Texas MD Anderson Cancer Center MD Anderson PSC, Houston, Texas, United States<br>• Novartis Investigative Site, Melbourne, Victoria, Australia<br>• Novartis Investigative Site, Toronto, Ontario, Canada<br>• Novartis Investigative Site, Montreal, Quebec, Canada<br>• Novartis Investigative Site, Chuo ku, Tokyo, Japan<br>• Novartis Investigative Site, Singapore, Singapore<br>• Novartis Investigative Site, Valencia, Comunidad Valenciana, Spain<br>• Novartis Investigative Site, Madrid, Spain<br>• Novartis Investigative Site, Sutton, Surrey, United Kingdom
15	Safety and Activity Study of PSCA-Targeted CAR-T Cells (BPX-601) in Subjects With Selected Advanced Solid Tumors	Recruiting	No Results Available	• Metastatic Castration-resistant Prostate Cancer<br>• Metastatic Prostate Cancer<br>• Metastatic Pancreatic Ductal Adenocarcinoma<br>• Metastatic Pancreatic Cancer<br>• Metastatic Pancreatic Adenocarcinoma	• Biological: BPX-601<br>• Drug: Rimiducid	• Moffitt Cancer Center, Tampa, Florida, United States<br>• Emory Winship Cancer Institute, Atlanta, Georgia, United States<br>• John Theurer Cancer Center, Hackensack University Medical Center, Hackensack, New Jersey, United States<br>• Columbia University Medical Center, New York, New York, United States<br>• Tennessee Oncology, PLLC, Nashville, Tennessee, United States<br>• Baylor Sammons Cancer Center, Dallas, Texas, United States<br>• The University of Texas MD Anderson Cancer Center, Houston, Texas, United States
